# Merging clinical chemistry biomarker data with a COPD database - building a clinical infrastructure for proteomic studies

**DOI:** 10.1186/s12953-017-0116-2

**Published:** 2017-04-21

**Authors:** Jonatan Eriksson, Simone Andersson, Roger Appelqvist, Elisabet Wieslander, Mikael Truedsson, May Bugge, Johan Malm, Magnus Dahlbäck, Bo Andersson, Thomas E. Fehniger, György Marko-Varga

**Affiliations:** 1 Centre of Excellence in Biological and Medical Mass Spectrometry, Biomedical Centre D13, Lund University, 221 84 Lund, Sweden; 2 Encap Security, Øvre Slottsgate 7, 0157 Oslo, Norway; 3 Section for Clinical Chemistry, Department of Translational Medicine, Lund University, Skåne University Hospital Malmö, 205 02 Malmö, Sweden; 4 Clinical Protein Science & Imaging, Biomedical Centre, Department of Biomedical Engineering, Lund University, BMC D13, 221 84 Lund, Sweden; 5 Örestadskliniken, 217 67, Eddagatan 4, 217 67 Malmö, Sweden; 6 First Department of Surgery, Tokyo Medical University, 6-7-1 Nishishinjiku Shinjiku-ku, Tokyo, 160-0023 Japan

**Keywords:** Proteomics, COPD, Clinical study, Biomarkers, Proteomics, Biobanking, Bioinformatics, EDC

## Abstract

**Background:**

Data from biological samples and medical evaluations plays an essential part in clinical decision making. This data is equally important in clinical studies and it is critical to have an infrastructure that ensures that its quality is preserved throughout its entire lifetime. We are running a 5-year longitudinal clinical study, KOL-Örestad, with the objective to identify new COPD (Chronic Obstructive Pulmonary Disease) biomarkers in blood. In the study, clinical data and blood samples are collected from both private and public health-care institutions and stored at our research center in databases and biobanks, respectively. The blood is analyzed by Mass Spectrometry and the results from this analysis then linked to the clinical data.

**Method:**

We built an infrastructure that allows us to efficiently collect and analyze the data. We chose to use REDCap as the EDC (Electronic Data Capture) tool for the study due to its short setup-time, ease of use, and flexibility. REDCap allows users to easily design data collection modules based on existing templates. In addition, it provides two functions that allow users to import batches of data; through a web API (Application Programming Interface) as well as by uploading CSV-files (Comma Separated Values).

**Results:**

We created a software, DART (Data Rapid Translation), that translates our biomarker data into a format that fits REDCap's CSV-templates. In addition, DART is configurable to work with many other data formats as well. We use DART to import our clinical chemistry data to the REDCap database.

**Conclusion:**

We have shown that a powerful and internationally adopted EDC tool such as REDCap can be extended so that it can be used efficiently in proteomic studies. In our study, we accomplish this by using DART to translate our clinical chemistry data to a format that fits the templates of REDCap.

## Background

Today, the integration and compilation of the large volume of data generated from clinical studies is challenging. This data has to be available to be used in companion diagnostic and prognostic tests which in turn are crucial at every level of clinical decision making. A multi-fold of patient samples is stored in biobanks for use in future medical research projects that measure the quantitative and qualitative read-outs of gene and protein expression associated with disease processes (1–2). Furthermore, biobanking laws that regulate data processing, integration, traceability, and confidentiality are implemented on a national level.

In Sweden, all institutions storing biological samples and clinical data are required to declare their inventories to the National Board of Health and Welfare. Sweden had 651 biobanks registered with the Board in 2007, the majority deployed at University hospitals and Regional Medical Authorities. The samples come from many forms of specimen including tissues, cells, cell lines, genomic material (DNA), blood, blood-plasma, and urine.

Project organization is essential to face the challenges in clinical studies, i.e., patient recruitment, appointment scheduling, blood samples collection etc. Additionally, other parts of the study are also important, such as sample storage, analysis, evaluation of the results, and compilation of data into a useful database. The latter part of the study may continue for a long time after clinical data collection is completed which is why a high quality database is crucial.

REDCap is a web-based EDC (Electronic Data Capture) tool developed by Vanderbilt University, TN, USA first released in 2004 [1]. REDCap is a secure application designed to support data capture in research studies with a user-friendly interface for data entry, audit trial to track data manipulation and export procedures, import data from external sources, and export procedures for data download to common statistical packages. We selected REDCap as the EDC tool for the current clinical study due to a version controlled update of the software, a massive user support, easy export/import of data using CSV-files (Comma-Separated Values), a large community of users, and the possibility to easily design different data collection instruments for various types of data input, e.g., text and numerical values.

KOL-Örestad is a 5-year longitudinal clinical study created to identify new biomarkers of COPD (Chronic Obstructive Pulmonary Disease) in blood. The biomarkers can be used to *diagnose* the disease, *predict* exacerbations, and to *classify* disease stages of COPD [2]. An early diagnosis would be beneficial to the patient, health-care system, and society due to lower medical costs and reduced sick leave. In the present study we describe how to transfer clinical chemistry data from the hospital information system to the KOL-Örestad database with a simple and secure EDC solution. Throughout the course of the study, blood samples are drawn from the participants at a private health-care clinic (Örestadskliniken), encoded, and sent to a public clinical chemistry laboratory. The analysis of these samples results in large number of numerical data values that need to be imported into the REDCap database. To enter that amount of data manually is time-consuming and error-prone. Instead, taking an approach that is automatic and indifferent to variable identifiers and order of variables is preferred.

In proteomic research it is critical that sample quality is preserved during storage, that sample inventories are maintained, and that the data produced by mass spectrometry or similar techniques is readily available for statistical analysis. This puts requirements on both the biobanks the samples are stored in as well as on inventory software and EDC tools.

## Methods

### Clinical study outline

The study group consists of approximately 300 study participants between the ages of 35 and 80 years. Two-hundred are diagnosed with COPD of one of the four stages of GOLD and 100 are healthy with normal lung function, both smokers/ex-smokers (*n* = 50) and never-smokers (*n* = 50). Participants undergo health examinations and blood sample collection every 6 months for a total of 5 years. Health examination includes a spirometer test, physical examination, and questionnaire [2]. Because the old classification system was in use at the initiation of the study, we will continue to use GOLD 1 to 4 based solely on post bronchodilator spirometry values.

Three 5 mL tubes of blood are sampled and analyzed for a set of predetermined disease biomarkers at a standard clinical chemistry laboratory and four tubes are aliquoted to 70 μL samples (plasma, serum, & whole blood) and stored in a biobank at -80^o^ C for protein biomarker analysis by mass spectrometry [3–8]. Each aliquot has a unique 2D barcode which may be traced back to the participant. The study has been approved by the regional ethical review board in Lund (DRN 2013/480) and is registered at ClinicalTrials.gov under the official title Biomarkers of Early Chronic Obstructive Pulmonary Disease (COPD) in Smokers - Longitudinal Study (U.S. National Institutes of Health, 2014).

###  Data sources

The study consists of different data input sources where each source has multiple variables of data that are to be stored in the same database. One data source is the primary health-care clinic where all visits are made. At each visit, the study participants fill out a questionnaire by hand and the responses are entered manually into REDCap by members of the research team. Additionally, the results from the physical examination and spirometry test are also entered manually into REDCap. The tubes from blood collection are labeled with barcodes identifying the study participant with a specific code and are entered into REDCap by a barcode scanner. The data is coded by a unique identifier (Study ID) for each participant and the full identification is solely available to the health-care personnel. However, full traceability from sample to Study ID will be accessible within the system.

### Database structure

The data is entered into REDCap at different locations and time points. Some data is entered manually and some is entered using the import function of REDCap. Preferably, all data should be entered automatically or electronically, e.g., through an automated direct input, digital scanning of the paper format, or import file from a data generating source. Currently, this is not possible in the present study; however, this is our goal.

### Data import to REDCap

The web interface of REDCap provides an import module that screens the data for errors before committing it to the database; e.g., errors such as incorrectly formatted data values or numerical values that lie beyond pre-defined bounds can be caught at this stage. Both batches of data as well as single data values can be imported through this module. Data batches are imported from CSV files whose format is required to match that which REDCap expects. Consequently, when an instrument generates a CSV files containing biomarker data it has to be translated, either manually or automatically, to this expected format. In addition to the import functions of the web user-interface, REDCap provides a web API that allows users to programmatically import and retrieve data to and from REDCap through HTTP methods. Lastly, the back-end of REDCap, a MySQL database, can be directly modified; however, this bypasses the above mentioned data screening [1].

## Results

**Fig. 1:**
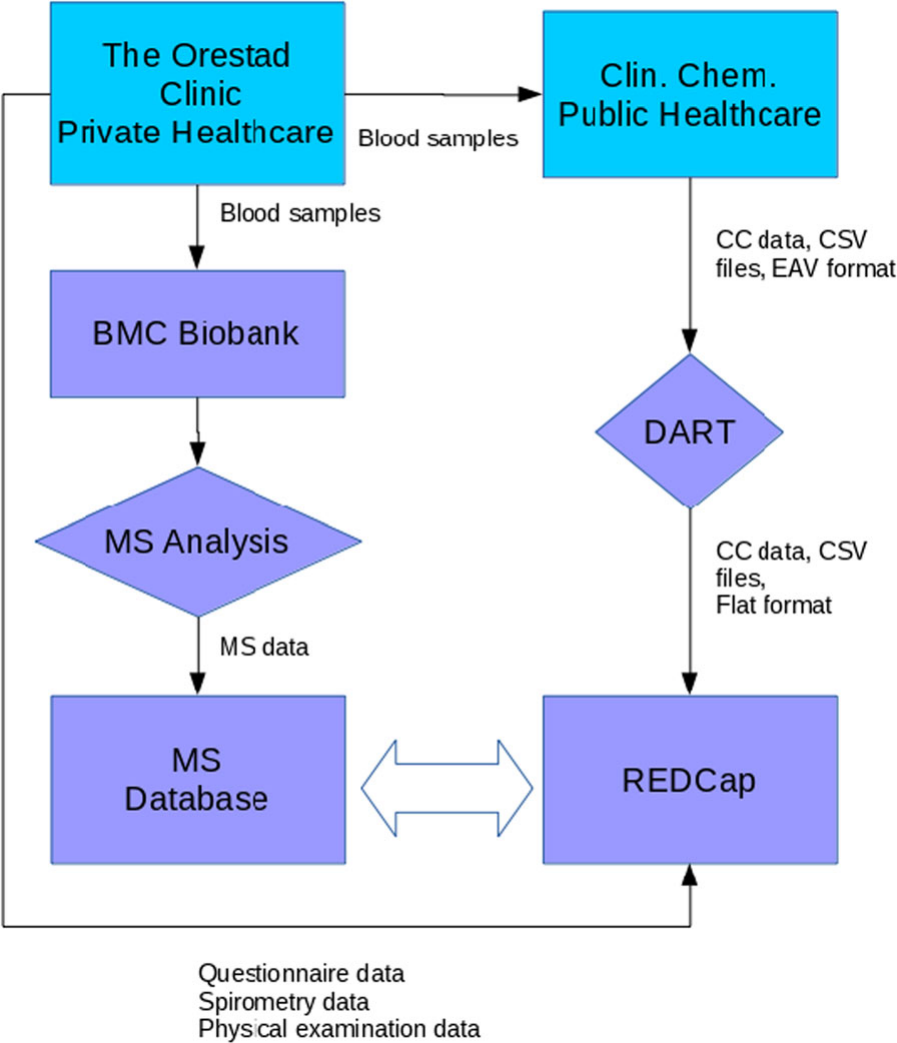
The sample and data flow of the study. Blood samples are drawn, physical examinations are performed, and health questionnaires are filled out at the health-care center. Blood samples are sent to the Clinical Chemistry center for analysis. Blood samples are also aliquoted and analyzed for protein biomarkers by SRM LC-MS/MS at the research center. The patient identifying data is stored in the same database as the rest of the data but is solely accessible to personnel at the health-care clinic

The study participants have their blood drawn, fill out questionnaires, and undergo physical examinations at the private health-care clinic. Members of the research team enter data from questionnaires and physical examinations manually into REDCap. The blood samples from each participant are sent both to BMC to be stored in a biobank and to a clinical chemistry facility in Malmö for disease biomarker analysis. The data generated by the biomarker analysis is stored in REDCap. Mass spectrometry (MS) analysis is performed on the biobank samples and the produced data is stored in a separate, dedicated, database. Results from analysis of the raw MS data, e.g., PSMs (Peptide-to-Spectrum Matches) or FDRs (False Discovery Rates), as well as data related to the experimental setup can also be stored in REDCap. The flow of samples and data can be viewed in Fig. 1. The data collected within the study, the data formats, and the input methods can be viewed in Table 1.

**Table 1:** Current data collection instruments, data format and input method to the KOL-Örestad database.

Source	Format	Input method
Blood samples	number, text, date, check box	manual, barcode scanner
Physical examination	numerical value	manual input from patient journal
Spirometry	number	manual input from paper
Questionnaire	number, text, check box	manual input from paper
CC analysis	number	import from data file
MS analysis	number, text	import from data file

The qualitative and quantitative output of the MS analysis (as well as its corresponding meta-data, e.g., MS platform, instrument setttings, Sequence Database, etc.) is stored in REDCap whereas instrument raw files are stored in a separate database

At the start of the study all data was imported manually into REDCap through its web interface. We chose to focus on automating the clinical chemistry data (CC data) importation since it involves the largest volume of data. The clinical chemistry laboratory instruments used to perform the biomarker analysis in the study automatically generate CSV files containing CC data. These files are formatted differently than those exported from and imported to REDCap. The instrument-generated files represent the data according to the EAV(Entity-Attribute-Value) model whereas the REDCap files mirror the flat table representation of the *DATA* table of the REDCap MySQL back-end. Furthermore, if the attribute and entity identifiers differ between the instrument and REDCap files the instrument files are not accepted by the batch import function of REDCap [1]. We created a program, DART (Data Rapid Translation), that automates the translation between the two formats. It extracts the biomarker data from the instrument-generated CSV file, re-formats it, and inserts it into the CSV file exported from REDCap. It does this by first translating the instrument-generated file to an intermediate hierarchical format which subsequently is translated to the format REDCap expects. DART is not exclusive to our particular pair of CSV files but can be configured to work with others as well.

DART was created using the C++ programming language. A minimal Graphical User Interface (GUI) was created using the wxWidgets library that allows the users to configure DART for different export/import formats, i.e., what the hierarchical structures look like and the mapping of the variable identifiers. DART is currently used in the study.

**Fig. 2:**
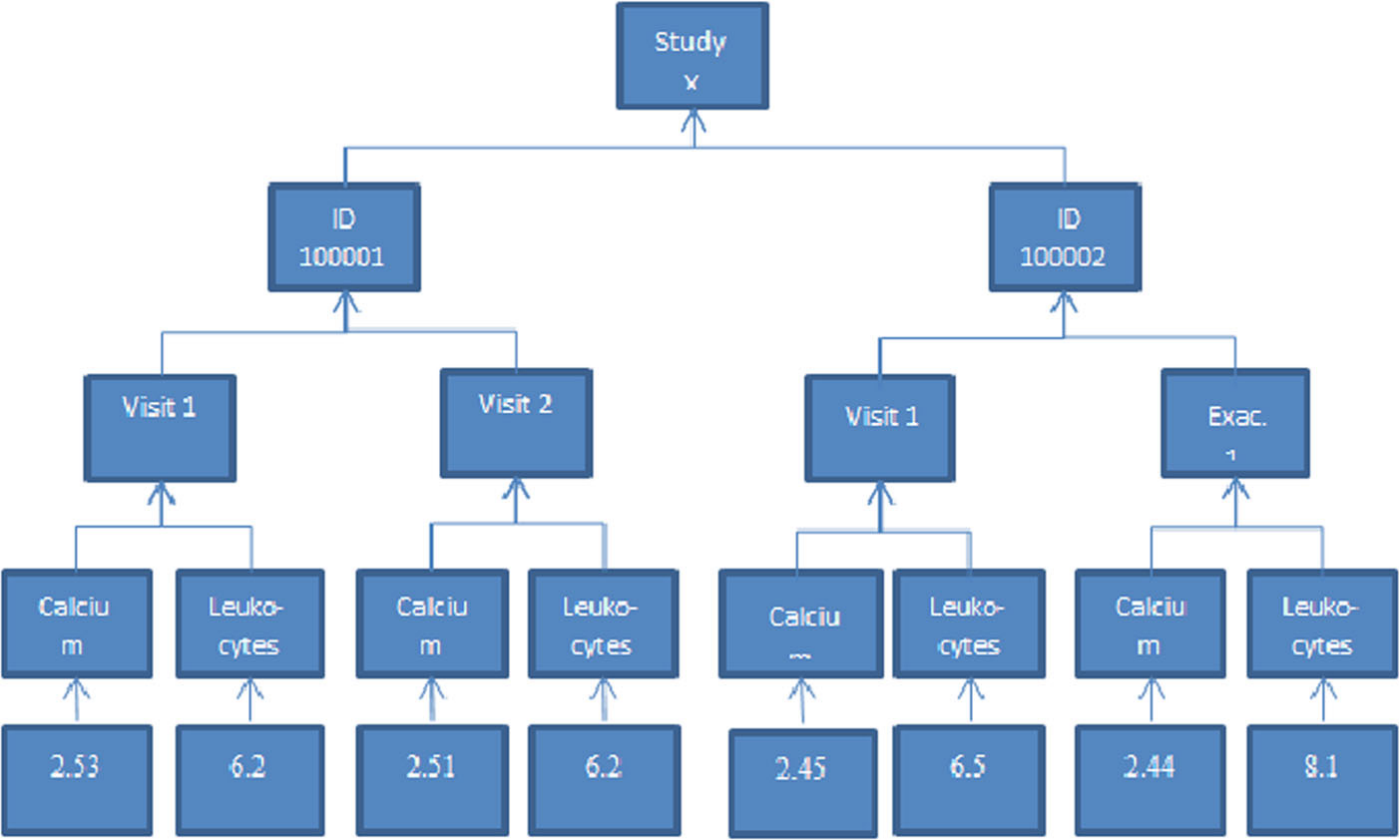
The hierarchy of the example data

An example of biomarker data and how it can be represented in a CSV file is shown in Table 2. The corresponding REDCap CSV file is shown in Table 3. The list of disease biomarkers used in Tables 2 and 3 is truncated. At the five levels of the hierarchy of the example data are, from top to bottom; the study id, the study participants, the visits as well as exacerbations of the participants, the biomarkers, and the numerical data value belonging to each biomarker. The hierarchy of the example biomarker data is illustrated in Fig. 2. The variables are not required to have the same identifiers in the REDCap and biomarker CSV files. The hierarchy is inherently the same in the file exported from REDCap, only formatted differently. Granted that the hierarchy of the data, the relationship between the format of the biomarker file and that of the file exported from REDCap, and the mapping of variable identifiers are specified, DART can transfer the data.

**Table 2:** Example of laboratory instrument output, the data is modeled as EAV

pat_id	visit_id	exacerbation_id	Date	Attribute	Value
100001	visit_1		150321	calcium	6.2
100001	visit_1		150321	leukocytes	6.5
100002	visit_1		150321	calcium	2.45
100002	visit_1		150321	leukocytes	6.5
100001	visit_2		150917	calcium	2.51
100001	visit_2		150917	leukocytes	6.2
100002		exacerbation_1	150613	calcium	2.44
100002		exacerbation_1	150613	leukocytes	8.1

**Table 3:** Example of a flat table exported from the REDCap database with biomarker data yet to be entered

patient_id	redcap_event_name	N	ca	leuco	hb	mono
100001	visit_1					
100002	visit_1					
100001	visit_2					
100002	exacerbation_1

## Discussion

In Sweden, there is no standardized way to manage and transfer clinical data. This makes it desirable to use an already adopted and well tested tool. The decision to use REDCap as the primary data management tool in this study was motivated by its accessibility, due to a simple web interface, to a broad user spectrum. It is used globally, particularly by non-profit research organization, in clinical studies [9]. The chosen approach where the database is exported as a CSV file, modified, and then imported back again, does not interfere with the built-in quality-control of data that REDCap provides. The alternative approach where the underlying database of REDCap is directly modified, while more straight-forward, circumvents this quality-control, and is thus more unsafe with respect to data integrity.

The combination of REDCap and DART is a flexible tool for the management of clinical study data. Some initial work has to be done when configuring the program for a particular import/export pair. Currently DART is solely used to import data into REDCap, it could, however, in a future iteration, also perform other tasks such as data analysis and/or presentation of data.

## Conclusion

It is crucial to store both data from sample analyses as well as the biological samples themselves in an organized and secure way during clinical studies. Furthermore, the data and samples have to be readily accessible to researchers and health-care personnel. In the KOL-Örestad study we achieve this by using REDCap to manage and store the data and our biobank to store the samples and their aliquots.

## Abbreviations

API: Application programming interface
COPD: Chronic obstructive pulmonary disease
CSV: Comma separated values
DART: Data rapid translation
EDC: Electronic data capture
GOLD: Global initiative for chronic obstructive lung disease
GUI: Graphical user interface

## References

[1] Marko-Varga G, Boja ES, Rodriguez H, Baker M, Fehniger TE (2014). J. Proteome Res, accepted *“*Biorepository Regulatory Frameworks: Building Parallel Resources that both Promote Scientific Investigation and Protect Human Subjects ”. J Proteome Res.

[2] Khleif SN, Doroshow JH, Hait WN (2010). AACR-FDA-NCI Cancer Biomarkers Collaborative Consensus Report: advancing the use of biomarkers in cancer drug development. Clin Cancer Res.

[3] Harris PA, Taylor R, Thielke R, Payne J, Gonzalez N, Conde JG (2009). Research electronic data capture (REDCap) - A metadata-driven methodology and workflow process for providing translational research informatics support. J Biomed Inform.

[4] Mikael T, Johan M, Barbara Sahlin K, May B, Elisabet W, Magnus D, Roger A, Fehniger TE, György M-V (2016). Biomarkers of early chronic obstructive pulmonary disease (COPD) in smokers and former smokers. Protocol of a longitudinal study. Clin Trans Med.

[5] Malm J, Fehniger TE, Danmyr P, Végvári A, Welinder C, Lindberg H (2013). Biobanking work flow standardization-developments providing sample integrity. J Proteomics.

[6] Malm J, Danmyr P, Nilsson R, Appelqvist R, Végvári A, Marko-Varga G (2013). Blood sample standardization developments for large scale biobanking. J Proteome Res.

[7] Fehniger TE, Boja ES, Rodriguez H, Baker M, Marko-Varga G (2014). Four areas of engagement requiring strengthening in modern proteomics today. J Proteome Res.

[8] Malm J, Linberg H, Erlinge D, Appelqvist R, Yokaleva M, Welinder C (2015). Semi-automated biobank sample processing with 384 high density sample tube robot used in cancer and cardiovascular studies. Clin Transl Med.

[9] Franklin J, Guidry A, Brinkley J (2011). A Partnership approach for Electronic Data Capture in small-scale clinical trials. J Biomedical Informatics.

